# Quantification and Comparison of Droplet Formation During Endoscopic and Microscopic Ear Surgery: A Cadaveric Model

**DOI:** 10.1177/0194599820970506

**Published:** 2020-11-03

**Authors:** Lukas Anschuetz, Abraam Yacoub, Tobias Buetzer, Ignacio J. Fernandez, Wilhelm Wimmer, Marco Caversaccio

**Affiliations:** 1 Department of Otorhinolaryngology–Head and Neck Surgery, Inselspital, University Hospital and University of Bern, Bern, Switzerland; 2 Hearing Research Laboratory, ARTORG Center for Biomedical Engineering, University of Bern, Bern, Switzerland; 3 Department of Otorhinolaryngology–Head and Neck Surgery, Faculty of Medicine, Ain Shams University, Cairo, Egypt; 4 Department of Otorhinolaryngology–Head and Neck Surgery, University Hospital of Modena, Modena, Italy

**Keywords:** endoscopic ear surgery, COVID-19, epitympanectomy, mastoidectomy, cholesteatoma, aerosol, safety

## Abstract

**Objectives:**

The COVID-19 pandemic and the disproportional spread of the disease among otorhinolaryngologists raised concerns regarding the safety of health care staff. Therefore, a quantitative risk assessment for otologic surgery would be desirable. This study aims to quantitatively compare the risk of perioperative droplet formation between microscopic and endoscopic approaches.

**Study Design:**

Experimental research.

**Setting:**

Temporal bone laboratory.

**Methods:**

The middle ear of whole head specimens was injected with fluorescein (0.2 mg/10 mL) before endoscopic and microscopic epitympanectomy and mastoidectomy. Fluorescent droplet deposition on the surgical table was recorded under ultraviolet light, quantified, and compared among the interventions. Drilling time, droplet proportion, fluorescein intensity, and droplet size were assessed for every procedure.

**Results:**

A total of 12 procedures were performed: 4 endoscopic epitympanectomies, 4 microscopic epitympanectomies, and 4 mastoidectomies. The mean (SD) proportion of fluorescein droplets was 0.14‰ (0.10‰) for endoscopic epitympanectomy and 0.64‰ (0.31‰) for microscopic epitympanectomy. During mastoidectomy, the deposition of droplets was 8.77‰ (6.71‰). Statistical comparison based on a mixed effects model revealed a significant increase (0.50‰) in droplet deposition during microscopic epitympanectomy as compared with endoscopic epitympanectomy (95% CI, 0.16‰ to 0.84‰).

**Conclusions:**

There is considerable droplet generation during otologic surgery, and this represents a risk for the spread of airborne infectious diseases. The endoscopic technique offers the lowest risk of droplet formation as compared with microscopic approaches, with a significant 4.5-fold reduction of droplets between endoscopic and microscopic epitympanectomy and a 62-fold reduction between endoscopic epitympanectomy and cortical mastoidectomy.

The rapid global spread of COVID-19 resulting from the novel coronavirus strain SARS-CoV-2 forced the World Health Organization to classify it as a pandemic on March 11, 2020.^1^ This current outbreak has raised concerns about the substantial risk of transmission of airborne infectious diseases among health care professionals and the best protective practices to avoid it. Early reports from China have stated that among health care professionals, otorhinolaryngologists were more vulnerable to infection than other colleagues in the same hospital. These infections are probably due to close contact with the high viral-loaded upper respiratory mucosa of infected patients.^2,3^ These alarming observations have elicited critical questions about the safety of outpatient and operating procedures.

On April 1, 2020, the US National Academies of Science, Engineering and Medicine reported that COVID-19 is likely to be transmitted via aerosols.^4^ The published letter cited a study carried out at the University of Nebraska Medical Center, which stated that SARS-CoV-2 RNA was identified in air samples taken from the hospital rooms of infected patients.^5^ A case report on COVID-19 infection transmitted to 14 Chinese health care professionals after a transnasal pituitary adenoma surgical procedure identified the probable infection route as postoperative, as medical staff outside the operative room were infected whereas those participating in the surgery were not.^6^ These findings clearly demonstrate the importance of adequate protective equipment. In routine otolaryngologic operative practice, many procedures are considered to be aerosol generating, such as tracheostomy,^7^ endoscopic sinus surgery,^8^ and mastoidectomy.^9^

As recently published, middle ear and mastoid mucosal linings are involved by SARS-CoV-2.^10^ Moreover, previous studies have detected other coronaviruses in the middle ear fluid of patients affected with otitis media.^11^ Given the infectious risk of contaminated middle ear fluids, it must be borne in mind that the use of powered instruments is a source of dispersion of droplets throughout the operative field. In light of this, transcanal endoscopic middle ear procedures are probably a less risky approach than conventional microscopic techniques, particularly since the external auditory canal acts as a natural protective shield from the droplets generated during surgical procedures. The aim of the current study was to simulate droplet generation during endoscopic and microscopic procedures with powered instruments and to quantify the droplet formation.

## Materials and Methods

### Ethical Issues

The institutional review board (Kantonale Ethikkomission Bern) granted approval to perform the present study (KEK-BE 2016-00887).

### Study Setup

**Figure 1.**
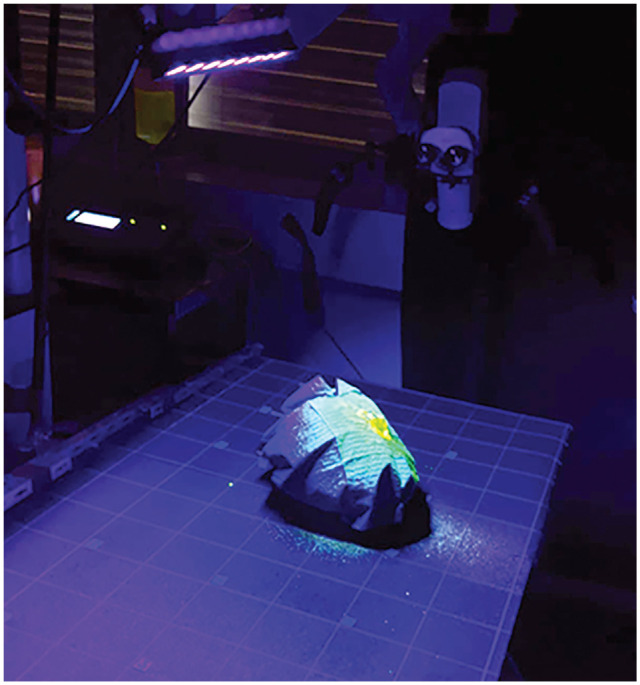
Study setup. A black mat with grid lines is placed on the surgical table, and an ultraviolet lamp is fixed to the ceiling. The whole head preparation was positioned in the middle of the table.

A surgical table was covered with a 200 × 100–cm black mat and divided into 10 × 10–cm rectangles with removable white grid lines. The rectangles (subquadrants) were consecutively numbered with letters for rows and numbers for lines, and 4 rectangles were considered a quadrant, as spread over a surface of 100 × 60 cm. A 24-W ultraviolet (UV) light source (BUV93; BeamZ) was fixed to the ceiling at a distance of 120 cm above the dissection table, and the temporal bone dissection laboratory was completely shaded from daylight. Thereafter, the whole head specimen was tightly wrapped with sterile drapes, leaving the ear free, and then placed in the middle of the operating field. The study setup is illustrated in **Figure 1**.

### Surgical Procedures

Adequate protective equipment was worn by the experimental team at all times. The endoscopic procedures were performed with endoscopes (14 cm long, 3-mm diameter) attached to a high-definition camera system and screen (Karl Storz). After elevation of the tympanomeatal flap, the middle ear was injected with a fluorescein solution (0.2 mg/10 mL of saline solution). Thereafter, the epitympanum was resected with a 3-mm coarse diamond drill (Bien Air Surgery). After suctioning of debris and fluorescein solution, the middle ear was again injected with fluorescein. Drilling continued until the whole body of the incus was visible.

Similarly, a microscopic epitympanectomy was performed with a surgical microscope (Leica) via a standard retroauricular approach. The skin was retracted with hook retractors fixed to the drapes. The simulation was completed by performing a cortical mastoidectomy under microscopic view with standard cutting burs.

### Measurements

Quantification of droplet formation was by measurement of fluorescein droplet deposition on the black surgical table. Pictures were taken under UV light with a camera (Nikon D3) at a predefined and constant height of 45 cm over the surgical table. Each quadrant (A1-C5) was photographed separately before and after every surgical procedure. Between the procedures, the grid lines were removed and cleaned with 80% ethanol, as was the surgical table. The cleaning was visually controlled under UV light.

### Image Processing and Quantitative Analysis

The image-processing steps for each quadrant were as follows:

1. Orthorectification and cropping of the photographs to the area of the quadrant to eliminate perspective distortion (Perspective Rectifier; RectifierSoft)
2. Calculation of the difference image (by subtraction of the presurgery photograph) to remove the background
3. Isolation of green-channel pixel values above a selected intensity threshold (64/255) to identify fluorescein-covered areas
4. Droplet detection
5. Identification and removal of grid lines

Steps 2 to 5 were executed with the Image Processing Toolbox of MATLAB 2016a (MathWorks). Quadrants containing the specimen (n = 24) and quadrants with defects (large drops of fluorescein; eg, from aspirator or drill) and blurred photographs (n = 23) were excluded, leaving 133 quadrants for analysis.

The processed quadrant images were combined into 1 overall image for each procedure. For each overall image, the following outcome measures were calculated:

*Droplet proportion*: proportion of fluorescein-covered area in per mille (‰; number of green pixels [representing fluorescein-covered areas] divided by total number of pixels)
*Median intensity*: median intensity of fluorescence, represented as green values above the intensity threshold (64/255)
*Median droplet size*: median droplet diameter in millimeters (median number of adjacent green pixels scaled to the image dimensions)
*Maximum droplet size*: maximum droplet diameter in millimeters (maximum number of adjacent green pixels scaled to the image dimensions)

The mean overall value and standard deviation were calculated for each procedure and outcome measure.

### Statistical Analysis

Separate general linear mixed models were used to examine the effect of the endoscopic and microscopic epitympanectomy (fixed factor, 2 levels) for each outcome measure. The specimen ID was used as the random intercept to account for repeated measures. Data were analyzed with MATLAB 2016a. Due to the different nature of the procedure, mastoidectomy was not considered in the comparative statistical analysis.

## Results

**Figure 2.**
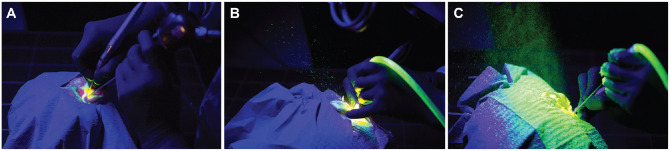
Snapshots of aerosolization risk per surgical technique: (A) endoscopic epitympanectomy, (B) microscopic epitympanectomy, and (C) mastoidectomy.

**Figure 3.**
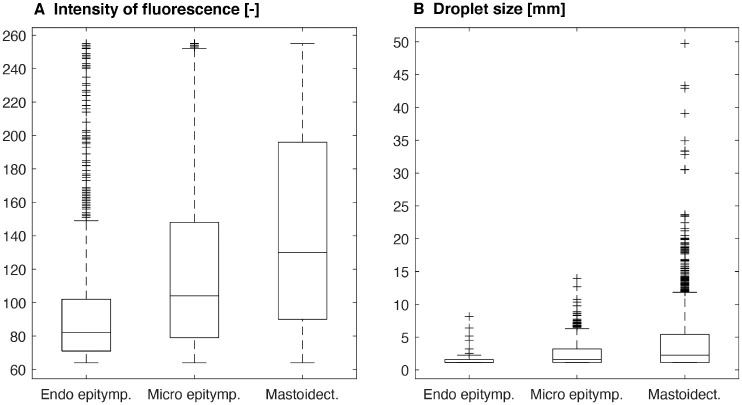
(A) Fluorescein intensity (pixel value up to 255) and (B) droplet size per surgical intervention: endoscopic epitympanectomy, microscopic epitympanectomy, and mastoidectomy. Box indicates 50% of values, with the median as a horizontal line; whiskers indicate minimum and maximum values without outliers (plus symbols).

A total of 12 surgical procedures were performed and analyzed: 4 endoscopic epitympanectomies, 4 microscopic epitympanectomies, and 4 mastoidectomies. The mean pure drilling times for the procedures were similar: 4:02 minutes for endoscopic epitympanectomy, 3:49 minutes for microscopic epitympanectomy, and 3:56 minutes for mastoidectomy. The mean (SD) overall proportion of fluorescein droplets per surgical intervention was 0.14‰ (0.10‰) for endoscopic epitympanectomy and 0.64‰ (0.31‰) for microscopic epitympanectomy. During mastoidectomy, the deposition of droplets was 8.77‰ (6.71‰). Examples of the droplet spray generated during the surgical interventions are illustrated in **Figure 2**. The median intensity of fluorescence and the median droplet size on the surgical table are summarized in **Table 1** and **Figure 3**.

**Table 1.** Results of the Outcome Measures for Each Surgical Intervention.

	Intervention				
	1	2	3	4	Mean (SD)
Droplet proportion, ‰					
Endoscopic epitympanectomy	0.15	0.01	0.25	0.14	0.14 (0.10)
Microscopic epitympanectomy	0.22	0.84	0.89	0.62	0.64 (0.31)
Mastoidectomy	3.06	16.65	3.32	12.03	8.77 (6.71)
Median fluorescence intensity					
Endoscopic epitympanectomy	80	85	80	86	82.75 (3.20)
Microscopic epitympanectomy	105	97	107	114	105.75 (6.99)
Mastoidectomy	99	138	124	126	121.75 (16.38)
Median droplet size, mm					
Endoscopic epitympanectomy	0.08	0.08	0.08	0.08	0.08 (0.0)
Microscopic epitympanectomy	0.08	0.20	0.12	0.08	0.12 (0.06)
Mastoidectomy	0.17	0.14	0.25	0.14	0.18 (0.05)
Maximum droplet size, mm					
Endoscopic epitympanectomy	1.01	1.19	1.15	2.74	1.52 (0.81)
Microscopic epitympanectomy	1.60	1.45	2.23	1.67	1.74 (0.34)
Mastoidectomy	3.03	5.58	3.29	5.19	4.27 (1.30)

Microscopic epitympanectomy led to a statistically significant increase in the proportion of droplets by 0.50‰ as compared with endoscopic epitympanectomy (*P* = .01; 95% CI, 0.16‰ to 0.84‰), and the median intensity increased significantly by 23.00 (*P* < .001; 95% CI, 15.12 to 30.88). No statistically significant differences were observed regarding median droplet size (*P* = .16; 95% CI, –0.02 to 0.10) and maximum droplet size (*P* = .60; 95% CI, –0.72 to 1.15) between microscopic and endoscopic epitympanectomy.

**Figure 4.**
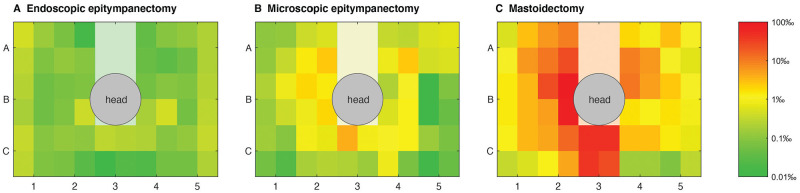
Proportion of fluorescent droplet deposition for each procedure as a logarithmic color-coded scale on the surgical table. The gray and white areas indicate the position of the head and sterile drapes.

The distribution of droplets in the different quadrants (A1-C5) was not homogeneous in the surgical field. More droplets were present on the left side of the specimen, since all of the surgeons performing the procedures were right-handed. The average distribution of droplets among the quadrants in the surgical field is illustrated in **Figure 4**.

## Discussion

In this study, droplet formation and the subsequent deposition of middle ear fluid during the procedures involved in ear surgery were compared under standardized laboratory conditions. Our results indicate a statistically significant 4.5-fold reduction in droplet generation with the endoscopic technique as compared with the retroauricular microscopic technique for epitympanectomies. Moreover, a 62-fold reduction was observed between endoscopic epitympanectomy and mastoidectomy.

The pandemic spread of COVID-19 dramatically highlighted the danger of infectious diseases, especially when highly infectious and with airborne transmission. Due to the proximity to the patient’s head, the infectious mucosal secretions with high viral load, and the manipulations frequently required for diagnostic or therapeutic purposes, otolaryngologists faced a considerable ordeal during the actual pandemic. Early reports from China indicated a pattern of “overinfection rates” among otolaryngologists as compared with other medical specialties.^2,3^ Therefore, adequate management of nonemergency cases and especially the protection of surgeons and operating room personnel should be of the highest priority.^12,13^ Moreover, no reliable diagnostic tests are actually widely available, which may impede the reliability of preoperative testing. Additionally, a negative test may lead to decreased adherence to wear personal protective equipment by the medical staff with possible spread of the virus due to false-negative test results. It should also be considered that future pandemics may occur with potentially more aggressive infectious agents.

Endoscopic ear surgery has been developed in recent decades and has gradually gained in importance worldwide in the treatment of different middle ear pathologies^14-16^ and, more recently, in minimally invasive lateral skull base surgery.^17^ Moreover, the endoscopic approach allows the exploration of the middle ear and even hidden regions, generally with no need for any kind of canaloplasty.^18-21^ As indicated by the results presented in this study, the endoscopic approach also appears to be minimally invasive in regard to droplet formation despite the use of powered instruments. One reason is the natural corridor to the middle ear offered by the external auditory canal, acting as a protective shield against aerosol generation. Moreover, the “heads-up” position during endoscopic ear surgery may be more favorable to the wearing of personal protective equipment without disturbing the surgeon’s view of the operative field as compared with the microscopic approach (eg, face shields). Therefore, the use of an endoscopic approach to the middle ear may be advocated whenever the type and extent of the pathology allow it.

It must be strongly emphasized that with extensive spread of the disease (e.g. cholesteatoma) into the mastoid, a retroauricular approach with mastoidectomy may be mandatory to completely eradicate the disease. Moreover, the use of standard otologic tools such as the curette may be recommended, as lower droplet generation may be expected. However, this was not the subject of this study, and manual curetting of bone may be limited.

The rigorous and correct use of personal protective equipment is strongly recommended during a mandatory mastoidectomy.^12^ Strategies to mitigate aerosolization during mastoidectomy have recently been published.^22,23^ They generally consist of a protective shield mounted on the objective of the microscope, forming a tent-like retainer of fluids and particles generated during drilling. These innovative strategies should be applied to tackle the increase in aerosol generation during mastoidectomy as identified in this study. However, the use of a minimally invasive therapeutic strategy appears to be beneficial to the patient^24,25^ and may also increase the safety and well-being of the operating room personal.

The main limitation of this study is that the simulated surgical procedures were standardized to ensure reproducibility and comparability. However, the quantification of aerosolization depends, for example, on the use of powered instruments and the rotation speed of the drill. As the difference in aerosol generation among the techniques under the investigated and standardized conditions is considerable, we favor the protective effect of endoscopic ear surgery, despite the aforementioned limitations. However, the exact magnitude of droplet formation will vary as the parameters of the experimental setup are changed.

## Conclusions

During otologic surgery, the magnitude of droplet formation from middle ear fluids is considerable and represents a risk for spreading airborne infectious diseases. The endoscopic technique offers the lowest droplet generation when compared with microscopic approaches, with a significant 4.5-fold reduction in droplet generation between endoscopic and microscopic epitympanectomy and a 62-fold reduction between endoscopic epitympanectomy and cortical mastoidectomy.
